# Connexin expression patterns in diseased human corneas

**DOI:** 10.3892/etm.2014.1530

**Published:** 2014-02-10

**Authors:** JIAJIE ZHAI, QIN WANG, LIANG TAO

**Affiliations:** Department of Pharmacology, Zhongshan School of Medicine, Sun Yat-Sen University, Guangzhou, Guangdong 510040, P.R. China

**Keywords:** cornea, connexin, expression, localization

## Abstract

The present study aimed to explore the feasibility of using antisense connexin (Cx) treatment to promote corneal wound healing, and to investigate the changes of Cx gap junction proteins in terms of mRNA, protein expression and distribution in human corneas that were diseased due to various causes. A total of 13 diseased corneas were studied, which were obtained from five eyes injured by chemical burns, five infected eyes and three eyes with Stevens-Johnson syndrome (SJS)-affected corneas. Total RNA was extracted from the corneas and processed by qPCR with isoform primers to detect the expression of eight Cxs. Flow cytometry was adopted to determine the differences in the expression levels of Cx26, Cx31.1 and Cx43. Immunofluorescence was employed to show the localization of the three aforementioned Cxs. The qPCR results indicated that of the eight Cxs, only Cx26, Cx31.1 and Cx43 were upregulated in diseased corneas. Flow cytometry showed that all the diseased corneal tissues, with the exception of the SJS-affected corneas, showed a significantly higher percentage of cells that expressed Cx26 and Cx31.1 compared with the percentage in normal corneas (P<0.05). For Cx43, all three injured corneal groups showed a significantly higher percentage of cells that expressed Cx43 compared with the percentage in normal corneas (P<0.05). Immunohistochemical staining showed that the localization of Cx26, Cx31.1 and Cx43 differed between normal corneas and diseased corneas. This study elucidated the alteration of Cx expression patterns in several corneal diseases. The results indicated that Cx26, Cx31.1 and Cx43 are upregulated in chemically burned and infected corneas at the mRNA and protein levels, whereas only Cx43 is upregulated in SJS-affected corneas.

## Introduction

Connexins (Cxs) are a family of structurally-associated transmembrane proteins that assemble to form vertebrate gap junctions (1). They are classified according to their predicted molecular mass, for example, Cx26 for a predicted mass of 26kDa and Cx43 for a predicted mass of 43kDa. Gap junctions are intercellular channels that allow direct communication of ions, metabolites and secondary messengers between neighboring cells that are essential for a number of physiological processes (2). Cxs are assembled in groups of six to form hemichannels or connexons, and two hemichannels then combine to form a gap junction (3). Mutation in Cx expression often results in functional or developmental abnormalities.

Antisense oligodeoxynucleotides (AsODNs) are short synthetic analogs of natural nucleic acids designed to specifically bind to a target messenger RNA (mRNA) by Watson-Crick hybridization, resulting in selective degradation of the mRNA or prohibiting translation of the selected mRNA into protein. Antisense agents are being studied as treatments for numerous diseases known to be caused by particular genes. Antisense Cx43 and antisense Cx31.1 have been developed to modulate Cx43 expression at the cellular and tissue levels and have been identified to accelerate skin wound healing in various animal models and clinical trials (4). There are also studies that indicate antisense Cx43 and Cx31.1 are novel therapeutic candidates for ocular wounds, particularly corneal wounds (5,6).

The transparent cornea has a highly ordered structure that transmits light and serves as a barrier between external and intraocular environments. Once the cornea is injured, visual deterioration is likely to be the ultimate consequence. There are numerous causes of injury, the most commonly observed of which include infectious diseases, chemical burns and Stevens-Johnson syndrome (SJS). Corneal infectious diseases involve inflammation of the cornea resulting from infection by bacteria, fungi or viruses and are among the leading causes of blindness worldwide. Chemical injuries, such as those caused by acid and alkaline agents, may cause extensive damage resulting in visual impairment through corneal scarring, melting and necrosis (7–10). SJS is a rare, potentially life-threatening immune-complex hypersensitivity disorder triggered by medications or infections. Ocular surface involvement is present in 67–81% of patients and may be potentially blinding in severe cases (11,12). Severe dry eye, which ultimately results in corneal opacification and eyelid deformities, is the most common long-term ocular complication, which is found in 46% of patients (12). Corneal opacification significantly hampers vision and there may be subsequent epithelial defects, ulceration, inflammatory haze, neovascularization as well as limbal stem cell deficiency in severe cases (13). To the best of our knowledge, Cx expression patterns in diseased corneas have not been well studied. In order to further explore the feasibility of using antisense Cx treatment to improve corneal wound healing, changes in Cx gap junction protein in terms of mRNA and protein expression levels and distribution in human corneas injured by inflammation, chemical burns and SJS were investigated in the present study.

In this study, the mRNA levels of various Cxs in human cornea tissue with chemical burn, infectious diseases and SJS were first examined; flow cytometry and immunostaining were employed to study protein expression levels and localizations, in the hope of unraveling the potential roles of CXs in the pathophysiology of these diseases that result in corneal injury.

## Materials and methods

### Sample collection

Approval for all human tissue-based research was obtained from the ethics committee of Zhongshan Ophthalmic Center (Sun Yat-Sen University, Guangzhou, China). Written consent was obtained from every patient. Human tissue was handled according to the tenets of the Declaration of Helsinki. Human corneal buttons (n=18) were examined: Five normal corneas, which were not suitable for corneal transplantation, were obtained from the Eye Bank of Guangdong Province (Guangzhou, China); five chemically burned corneas, five inflammatory corneas and three corneas affected by SJS were obtained from patients who had undergone penetrating keratoplasty. The demographic and clinical characteristics of all donors of the diseased corneas are depicted in Table I. All corneas were divided into four quadrant segments; one quadrant for quantitative polymerase chain reaction (qPCR), the second quadrant for immunohistochemistry, the third quadrant for flow cytometry, while the fourth quadrant was stored at −80°C for further investigation.

### RNA extraction and qPCR

Total RNA was extracted using an RNeasy Micro kit according to the manufacturer’s instructions (Qiagen Inc., Valencia, CA, USA). DNase I (Qiagen Inc.) was used to exclude DNA contamination. The total isolated RNA was quantified by its absorption at 260 nm and then stored at 280°C until use. With the housekeeping gene, GADPH*,* as an internal control, the messenger RNA (mRNA) expression of eight Cx genes was analyzed in normal and diseased human corneas by qPCR, which was performed using ABI TaqMan MGB chemistries and an ABI Prism 7000 light thermocycler (Applied Biosystems, Foster City, CA, USA). Briefly, primers and TaqMan probes were designed using PrimerExpress software 4.0 (Applied Biosystems) and qPCR reactions were optimized to ensure high amplification efficiency (Table II). qPCR was established by terminating reactions at the intervals of 20, 24, 28, 32, 36 and 40 cycles for each primer pair to ensure that PCR products were within the linear portion of the amplification curve. All products were separated by 2% agarose gel electrophoresis and visualized with 0.5 mg/ml ethidium bromide. The fidelity of the qPCR products was verified by comparing their size with the size of cDNA bands and by sequencing the PCR products, and expression levels in the diseased cornea were normalized to the average level of respective mRNA in normal cornea. All reactions were performed in triplicate.

### Flow cytometry

All the samples used for flow cytometry were digested in 2 mg/ml collagenase type IV (Sigma-Aldrich, St. Louis, MO, USA) and 0.05 mg/ml DNase I in RPMI-1640 for 90 min at 37°C followed by trituration to form a single cell suspension. The suspension was then passed through a 40-μm cell strainer and washed in RPMI medium. Cell suspensions were incubated with antibodies against Cx26, Cx31.1 and Cx43 (affinity purified mouse monoclonal antibody; Sigma-Aldrich) in FACS buffer [phosphate-buffered saline (PBS), 2% fetal bovine serum and 0.1% sodium azide]. After staining, the cells were fixed in 1% paraformaldehyde and samples were analyzed by flow cytometry (FACS Aria; BD Bioscience, San Jose, CA, USA) with a 550-nm laser. Data were analyzed using Flowjo version 8.7.1 software (TreeStar, Ashland, OR, USA). Analyses were performed in triplicate and the results are displayed as the average expression percentage of total cell number.

### Immunofluorescence staining

Small tissue blocks (4×4 mm) from the central 6 mm of every cornea were frozen in liquid nitrogen and then transferred to a cryostat (Leica Jung CM 1500; Leica Microsystems, Wetzlar, Germany) and cut into quadrants; one quadrant was cut orthogonally to the corneal surface and mounted on an electrostatic slide (Superfrost Plus; Menzel-Gläser, Braunschweig, Germany). They were washed in PBS for 5 min prior to staining to remove optimal cutting temperature (OCT) medium. All slides were fixed in cold 99% methanol for 10 sec at −20°C, then washed three times for 5 min each in PBS and blocked with 10% fetal calf serum (FCS) for 30 min.

Immunofluorescence staining of the tissue was performed using a primary antibody against Cx26, Cx31.1 and Cx43 (Sigma-Aldrich). The primary antibody was diluted in 10% FCS (1:100) and sections were incubated overnight at 4°C. After rinsing the slides three times for 5 min each in PBS, the sections were incubated for 30 sec with the secondary antibody (goat anti-mouse Cy2-conjugated antibody; Jackson ImmunoResearch Laboratories, Inc., West Grove, PA, USA). Thereafter, slides were washed three times for 5 sec each in PBS and finally mounted in anti-fading solution for fluorescence microscopy (Mowiol; Merck KGaA, Darmstadt, Germany) containing DAPI (Sigma-Aldrich) and observed with the aid of a fluorescence microscope (Axiophot; Carl Zeiss Meditec AG, Oberkochen, Germany). Control slides underwent all the aforementioned procedures but without incubation with the primary antibody. Photography was achieved digitally with a camera attached to the microscope (AxioCam; Carl Zeiss Meditec AG). Multiple immunostaining experiments were performed on each cornea (at least four sets of independent experiments for each cornea). All parameters during image acquisition were kept constant throughout each experiment to allow direct comparison of all the 8-bit digital images. Expression levels of Cx proteins were quantified by counting Cx-positive pixels at the wound edges on binary images with identical thresholds using NIH ImageJ software (version 1.44 for Windows). Cx pixels were counted per micron squared of cornea in order to account for differences in corneal thickness.

### Statistical analysis

Statistical analysis was performed with SPSS for Windows, version 16.0 (SPSS, Inc., Chicago, IL, USA). The data of each group were compared and are expressed as the means ± standard deviation. P<0.05 was considered to indicate a statistically significant difference. Statistical comparisons between the groups were performed using analysis of variance.

## Results

### Cx mRNA expression in diseased corneas

**Figure 1:**
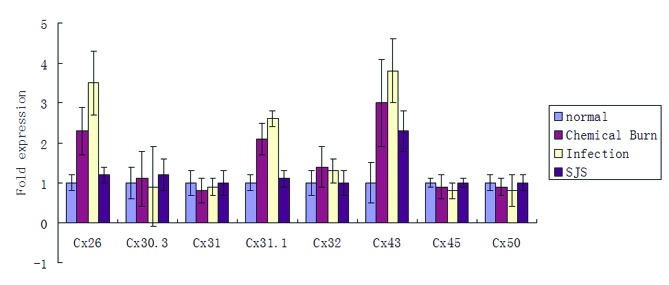
Quantitative polymerase chain reaction results of eight connexins in normal and diseased human corneas. ^*^P<0.05 vs. normal cornea. SJS, Stevens-Johnson syndrome.

qPCR was performed to evaluate the mRNA expression levels of Cxs. Of the eight cornea-associated Cx candidates, the mRNA levels of Cx26, Cx31.1 and Cx43 were significantly higher (P<0.05) in the diseased corneas than in the normal corneas (Fig. 1). The remaining five Cx candidates, Cx30.3, Cx31, Cx32, Cx45 and Cx50 were not observed to be significantly different between the diseased and the normal human corneas (Fig. 1).

### Flow cytometry

**Figure 2:**
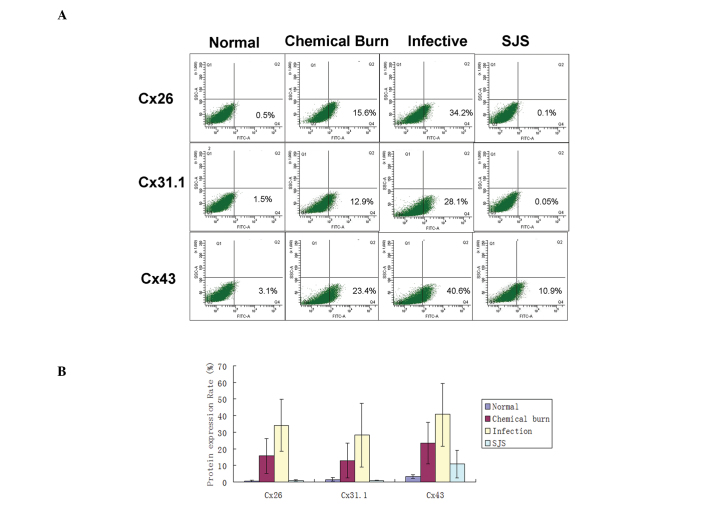
Quantitative analysis of the expression of Cx26, Cx31.1 and Cx43 in normal and diseased corneas by flow cytometry. (A) Flow cytometric analysis and (B) bar chart of protein expression levels. ^*^P<0.05 vs. normal cornea. SJS, Stevens-Johnson syndrome; Cx, connexin.

To further investigate the quantitative differences in Cx26, Cx31.1 and Cx43 expression levels, one quadrant of the diseased corneal tissue was evaluated by flow cytometry. As shown in Fig. 2, only 0.5±0.5% cells in normal corneal tissue expressed Cx26, but in the chemical burn, infected and SJS-affected groups, the percentages of cells that expressed Cx26 were 15.6±10.4, 34.2±15.6 and 0.1±0.06, respectively. In addition, with the exception of SJS-affected corneal tissue, all tissues showed a percentage of Cx26-expressing cells that was significantly higher compared with that of the normal corneas (P<0.05). For Cx31.1, the mean baseline percentage of expression was 1.5±1.2, and the percentages of Cx31.1 expressed in the chemically burned, infected and SJS-affected corneas were 12.9±10.6, 28.1±19.2 and 0.05±0.1, respectively. Chemically burned corneas and infected corneas had significantly higher percentages of Cx31.1-expressing cells compared with that of the normal corneas (P<0.05), while SJS-affected corneas did not show a significant difference from normal corneas. The baseline proportion of cells that expressed Cx43 was 3.1±1.1%. In the chemically burned, infected and SJS-affected corneas, the percentages of Cx43-expressing cells were 23.4±12.5, 40.6±19.1 and 10.9±8.4, respectively. In all three injured cornea groups, the percentages of cells that expressed Cx43 were significantly higher than the percentage in the normal corneas (P<0.05).

### Immunofluorescence staining

**Figure 3:**
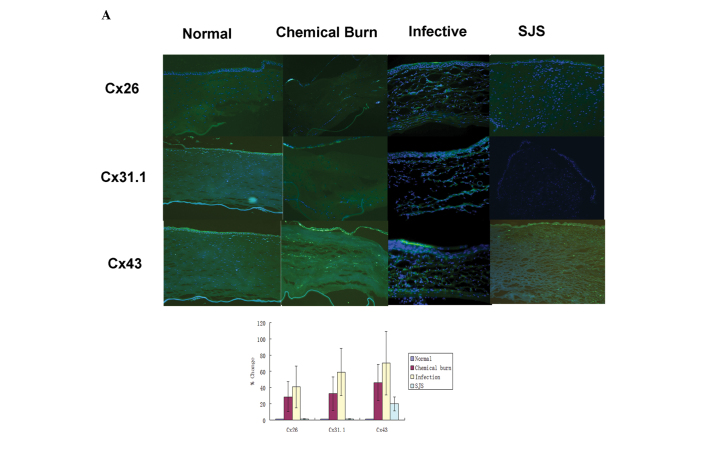

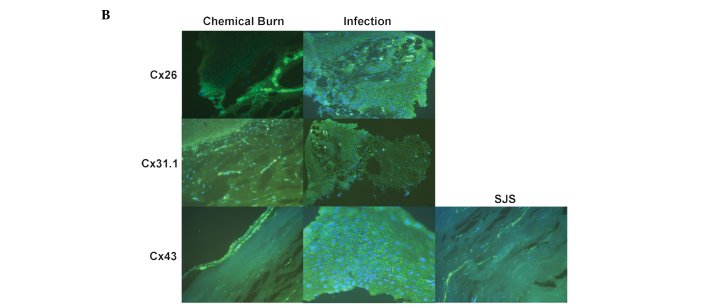
Immunohistochemical expression of Cx26, Cx31.1 and Cx43 in human corneas. Nuclei of corneal cells are stained blue (DAPI). Positive immunolabelling appears green (Cy2). (A) Expression of Cx26, Cx31.1 and Cx43 was quantified and the results are expressed in a bar graph. ^*^P<0.05 vs. normal cornea. Immunostaining for Cx26 and Cx31.1 was upregulated in chemically burned corneas, infected corneas and allograft failure corneas. Immunostaining for Cx43 was upregulated in all the diseased cornea groups. (A), magnification, ×100; (B), magnification, ×400. Cx, connexin; SJS, Stevens-Johnson syndrome.

Cx31.1 and Cx43 were mainly expressed in the corneal epithelium and the upper side of the corneal stroma in the normal corneas, whereas Cx26 showed marginal expression in the corneal epithelium. There were stronger expression levels of Cx26, Cx31.1 and Cx43 in the chemically burned corneas, but the distribution varied: Cx26 and Cx43 were mainly localized in the epithelium as well as the stroma, whereas Cx31.1 was mainly localized in the epithelium and the upper stroma. Infected corneas showed the strongest Cx26, Cx31.1 and Cx43 expression levels of all the corneal samples and expression was throughout the whole area. The infected corneas did not show a regular lamellar corneal shape due to the presence of ulcer lesions. In SJS-affected corneas, only the expression levels of Cx43 were increased in the corneal epithelium compared with those of the normal corneas; however, the expression levels of Cx26 and Cx31.1 were slightly downregulated compared with their levels in the normal cornea (Fig. 3).

## Discussion

Ocular trauma and corneal ulceration are significant causes of corneal blindness. These two causes are often under-reported but may be responsible for 1.5–2 million new cases of monocular blindness worldwide each year (14). When the cornea is wounded by chemicals, infection and hypersensitive immune responses (for example during the pathogenesis of SJS), the healing process starts immediately to re-establish corneal homeostasis by restoring the ocular surface permeability barrier function. Healing is achieved by the migration of adjacent cells to cover the injured area (4,15). The migrating cells undergo substantial phenotypic changes; these cells are then attached to the substratum as well as to each other. Thus, sufficient cell-to-cell contacts are retained to make sure the cells migrate as a cohesive sheet (16). One of the phenotypical attributes of the healing cornea is its gap junction-mediated (17) syncytial arrangement. Syncytial arrangements may be important in the coordination of physiological processes of tissue healing and regeneration. Also, it is becoming increasingly clear that Cxs have profound effects on gene expression during the process of development. Gap junction-deficient and -mutant mice present a range of debilitating phenotypes, such as hereditary diseases of the lens (zonular pulverulent cataract) (18), nervous system (X-linked Charcot-Marie-Tooth disease) (19), hereditary non-syndromic deafness (20), skin (palmoplantar keratoderma) (21), and bone and teeth (occulodigitoldental dysplasia) (22). These are additional reasons that altered expressions of gap junction proteins in injured human corneas require further investigation.

Cxs are the building units of gap junctions. In forming a gap junction, six Cxs oligomerize to form a hexameric hemichannel called a connexon. Two connexons join together to form a functional gap junction and direct cell-cell communication, namely paracrine signaling, is established. However, it should be noted that unpaired hemichannels also exist on the cell surface and play active roles in paracrine intercellular signaling, implicating the release of signaling molecules, such as ATP, from cells (23). These hemichannels constructed of Cxs are distributed around cells where their channels, when open, complete communication occurring directly across gap junctions.

Yuan *et al* (24) detected 10 Cx isoforms (Cx26, Cx30, Cx30.3, Cx31, Cx31.1, Cx32, Cx43, Cx45, Cx50 and Cx58) in the central and peripheral primate corneal epithelium by qPCR. Laux-Fenton *et al* (25) demonstrated that eight Cx transcripts (Cx26, Cx30.3, Cx31, Cx31.1, Cx33, Cx37, Cx43 and Cx50) were present in rat central cornea, and the peripheral cornea additionally expressed Cx30, Cx40, Cx45 and Cx46. In the present study, the mRNA expression levels of Cx26, Cx30.3, Cx31, Cx31.1, Cx32, Cx43, Cx45 and Cx50 of the normal and diseased human corneas were evaluated by qPCR. The expression of all eight Cx mRNAs was detected, which supported the results of Yuan *et al* and Laux-Fenton *et al*; however, only Cx26, Cx31.1 and Cx43 showed significant differences between diseased corneas and normal corneas. Therefore, the study focused on whether there were any alterations in the protein expression levels and the exact topographic distribution of these three Cx proteins in the diseased corneas.

Flow cytometry was employed to determine the Cx26, Cx31.1 and Cx43 expression levels. As the purpose of this study was to explore whether Cx proteins are upregulated or downregulated and to elucidate the role of antisense Cx treatments in modulating corneal wound healing, the cell types used in the flow cytometry experiments were not discriminated. Antisense Cx treatments are likely to be applied topically to the ocular surface *in vivo*; various cell types, such as corneal epithelial cells, corneal keratocytes and immune cells that have infiltrated during the diseased state are likely to be affected by the antisense treatments. The focus of the present study was the variation in protein expression levels of the specific Cx proteins in the overall diseased corneas. The results of flow cytometry supported the findings of the qPCR experiment, which indicated that Cx26, Cx31.1 and Cx43 mRNA levels as well as protein levels were upregulated in the chemically burned corneas and infected corneas, whereas for SJS-affected corneas, only Cx31.1 and Cx43 were upregulated; Cx26 did not show significant differences in protein levels from normal values.

Cx43 is the most predominant gap junction protein expressed in the basal layers of the corneal epithelium and in the anterior stroma (26,27). It contributes crucially to the regulation of corneal cell growth and differentiation, thus having a significant impact on the maintenance of corneal homeostasis (28,29). Ratkay-Traub *et al* (30) investigated the changes in Cx43 expression following excimer laser photorefractive keratectomy in rabbits and found that Cx43 expression was upregulated and relocated to the upper cell layers of the epithelium 24 h following surgery. Laux-Fenton (31) further examined the dynamics of Cx43 protein expression during normal corneal wound healing by using a rat scrape wound model and excimer laser surgery, and found downregulation of the Cx43 protein in the migrating epithelium at the wound front, but upregulation in the dividing epithelium further back from the wound leading edges. Cx43 was also upregulated in the stroma where it was involved in hypercellularity that ultimately resulted in corneal haze. In the present study, it was observed that in all the diseased human corneas the expression of Cx43 was upregulated both in the epithelium and stroma, which is similar to the results of Ratkay-Traub *et al* (30) and Laux-Fenton (31). The results of the present study further confirmed that the Cx43 expression patterns of animal models, which are usually employed to study the corneal wound healing process, are similar to that of human corneal diseases.

Recently, it was identified that in the rat corneal stroma following wounding, Cx43 protein is initially lost below the injury site as a result of cell dieback, but is subsequently upregulated in the stroma in the central and the peripheral areas of the wound site (32). In the present study, it was not possible to show the dynamic change in the expression of Cx43 in human corneal samples; however, it was demonstrated that Cx43 expression increases after corneal wounding. Patients with corneal chemical burns are not suitable for corneal transplantation surgery until the inflammatory reaction has ceased. In the samples collected in the present study, the mean surgery time was 0.5±0.3 years following the initial chemical burn accident, which means that the chemical burn samples were collected 0.5±0.3 years after the corneas were wounded, indicating Cx43 may have additional roles once the wound is healed with scarring. Further investigation in animal models is required to elucidate the dynamic changes of Cx43 mRNA and protein levels in the process of chemical wound healing. As for the patients with corneal infection, they underwent surgery as soon as they failed the medication treatments and their diseased corneas showed the strongest expression of Cx43 protein within all the groups. This result indicates that Cx43 is closely associated with corneal inflammation. It has been found that Cx43 is expressed in activated leukocytes and at leukocyte-leukocyte contact sites during their extravasations under inflammatory conditions. Additionally, functional Cx43 channels are involved in the release of cytokines and immunoglobulins (33). Therefore, the upregulated expression of Cx43 protein may be partly attributed to the infiltration of inflammatory cells into the diseased corneas. SJS is an immunological disease in which skin and mucous membranes are the primary target. Cx43 protein was upregulated compared with the normal corneal level by 10.9% in SJS-affected corneas, possibly as SJS-affected corneas are more prone to necrosis and have only minimal inflammation (34).

Cx31.1 is a relatively rare gap junction protein and appears to be unique in its inability to form functional gap junction channels, either with itself or with other Cx isoforms (35,36). It has been shown to be expressed in the middle and outer layers of the corneal epithelium in rat corneas, in suprabasal and superficial layers of epithelium as well as the upper parts of the cornea stroma (37). Expression of Cx31.1 in the corneal epithelium extends from the suprabasal layers of polyhedral wing cells through the flat squamous cells of superficial layers, which are shed into the tear film (31). Cx31.1 has also been shown to have a dynamic expression pattern during wound healing (38,39). Cx31.1-specific antisense oligodeoxynucleotides were used to evaluate its roles in a corneal epithelium model. Following antisense Cx31.1 treatment in rat and human corneal organotypic culture models, not only was there evidence for Cx31.1 knockdown, but also the cornea epithelium appeared significantly thicker within just 24 h and a marked reduction in epithelial apoptotic cell numbers was observed. The results indicated that Cx31.1 may play a role in triggering apoptosis, leading to cell sloughing into the tear film (6). In the current experiment, it was identified that Cx31.1 expression levels were significantly higher at the mRNA and protein levels in the chemically burned and infected corneas. These findings support the results of Chang *et al* (6)*,* as following chemical burns and infection, apoptosis is triggered both in corneal epithelial cells and corneal keratocytes (40). The present results, together with those of Chang *et al* further indicate that antisense Cx31.1 is likely to be a promising candidate for the treatment of corneal wounds.

Cx26 is normally expressed in the early embryonic proliferative epidermis. It is upregulated during wound re-epithelization and downregulated to low levels as terminal differentiation occurs to achieve the skin’s permeability barrier (41,42) Persistent inflammatory cell infiltration has been observed in re-epithelialized skin with high levels of Cx26, which may be due to impaired skin barrier function, Cx26 modulation or both (43). Djalilian *et al* suggested that decreasing the Cx26 levels of epithelialized skin lesions may encourage keratinocyte differentiation and immune response modulation in the skin (42). There are few publications about Cx26 in corneas in a pathological state; however, their distribution in normal corneas has been investigated (24,25). In the present study, Cx26 expression was shown to be upregulated at the mRNA and protein levels in chemically burned and infected corneas, which may have subsequently impaired the barrier function of these corneas.

In conclusion, the mRNA and protein levels of Cx31.1 and Cx43 were significantly upregulated in the human cornea samples affected by chemical burns or infection. The results further demonstrate the pathology of Cxs during the wound healing period and indicate that antisense Cx43 and Cx31.1 may be potential candidates for the treatment of chemically burned and infected corneas.

## Figures and Tables

**Table I:** Demographic and clinical data of the donors of the diseased corneas.

Disease	Patient no.	Age	Gender	PK	Cause
Chemical burn	1	23	F	L	Alcohol
	2	41	M	R	Hydrochloric acid
	3	36	M	R	Sodium hydroxide
	4	27	M	R	Hydrogen cylinder explosion
	5	50	M	L	Sulfuric acid
Infection	1	34	M	R	*Aspergillus flavus*
	2	54	M	L	*Pseudomonas aeruginosa*
	3	60	F	R	*Staphyloccocus aureus*
	4	25	F	R	Herpes simplex virus
	5	44	M	L	*Streptococcus pneumoniae*
SJS	1	14	M	L	Sulfonamide
	2	18	M	L	Diclofenac
	3	12	M	L	Sulfonamide

SJS, Stevens-Johnson syndrome; PK, penetrating keratoplasty.

**Table II:** PCR primers used.

Gene	Forward Primer (5′-3′)	Reverse Primer (5′-3′)	AT (°C)
Cx26	TCTTTTCCAGAGCAAACCGC	GACACGAAGATCAGCTGCAG	58
Cx30.3	GCTACCTGCTGCTGAAAGTC	CGTTGTGTATGAATGGAGCA	58
Cx31	GGCAAAGGATGAAAGCTCAG	CAACCACAGAGCGAGTGAAA	64
Cx31.1	GTGGACATATGTCTGCAGCC	CTATGAGAGATGCTAGAGC	60
Cx32	GCGAGGAGACAAGAGGAATG	AAGCAGCATGCAAATCACAG	64
Cx43	AACTGGCATTCTTGGGTTTG	CTCAGCATTTTCACCAGTCG	64
Cx45	GCACTGCCAGTAGCAAATCA	CCAACAGCATCCCTGAAGAT	62
Cx50	GGGCTACCAAGAGACACTGC	ACCTTCTCCTGCTCCTCCAT	64
*GAPDH*	ACCAAGATCATCCATGACAAC	GTCCACCACCCCGTTGCTGTA	64

Cx, connexin; AT, annealing temperature.
